# Optimal key forwarding strategy in QKD behaviours

**DOI:** 10.1038/s41598-024-64994-6

**Published:** 2024-06-17

**Authors:** Alin-Bogdan Popa, Pantelimon George Popescu

**Affiliations:** Computer Science and Engineering Department, National University of Science and Technology POLITEHNICA Bucharest, Bucharest, 060042 Romania

**Keywords:** Quantum information, Electrical and electronic engineering, Computer science

## Abstract

Nowadays QKD plays a critical role in unconditionally-secure and quantum-safe key distribution. Commercially available QKD devices are getting more popular for institutional and governmental national and international networks, but are expensive and offer limited key rates. We provide a formalization of QKD-generated key forwarding and redistribution at the KMS level by extending the network graph of physical QKD links to the complete graph with logical links, and we investigate its application on three practical scalable scenarios (all-to-all, one-to-all, one-to-one). We define a maximization goal for each scenario, and provide a linear programming problem statement to compute the optimal redistribution. We perform an extensive analysis of the algorithm in terms of forwarding results and key consumption on simulated QKD networks and discuss the implications of network size and graph topology on the algorithm’s performance and complexity.

## Introduction

A QKD network consists of a graph of nodes, each node representing a participant in the network (an end-user). The participants are connected through point-to-point QKD links (the edges of the graph). Each link connects two participants with a medium suitable for exchange of quantum information. In practice, the exchange of quantum information is typically performed via polarized photons, and the medium is typically fiber optics and/or free space (particularly when fiber optics are not available, for example in long distance communication, typically performed with satellites and optical ground stations). Both types of mediums can be used in a network at the same time (as is the case with the space-level extension of the EuroQCI network via ESA satellites, currently planned for 2026^1^). It is usual for a link between two end-users to consist of several segments due to practical distance constraints (for example, IDQuantique Cerberis XG requires a repeater segment once every 90 km^2^). However, since key re-distribution only concerns the actions of the end-users, we considered the edges of the graph as representing a link between two end-users and we ignored the practical realization of this links in terms of the physical layer and the number of repeater segments.

Each QKD link between two end-users Alice and Bob is composed of two QKD devices, one located on the premises of Alice and one located on the premises of Bob. The devices exchange quantum information to generate a series of pairs of identical bit sequences (keys), which can be accessed by both end-users and which are guaranteed to be unconditionally secure. The typical workflow is as follows: Alice requests a 256-bit key from her QKD device through the vendor-specific application-programming interface (API) built into the device; the API responds with the contents of the key and a public key ID; Alice sends this key ID to Bob over any public channel (for example, via Internet); Bob submits the key ID to his device’s API, which responds with a key identical to Alice’s. Now Alice and Bob hold the same key obtained in an unconditionally secure manner, which they can use to encrypt further communication.

The practical motivation for QKD infrastructure, several orders of magnitude more expensive than infrastructure for classical (non-quantum) key exchange algorithms over public channels, lies in the unconditional security of the generated keys and the guaranteed ability to withstand any future developments in cryptography algorithms that may be used in breaking it and in hardware capable of speeding up this process (such as, general use quantum computers capable of running Shor’s algorithm^3^). Unlike classical key exchange algorithms which can never be proven secure but rely on assumed security due to having withstood significant scrutiny and attempts to break it, QKD is proven to achieve perfect unconditional security and perfect forward secrecy in the key exchange mechanism.

The motivation for having these security properties obviously persists in the case of key exchange between end-users across multiple links. For example, a network may consist of three end-users: Alice (A), Bob (B), and Charlie (C), with QKD links between A–B and between B–C. A key forwarding mechanism implemented at Bob can be used to obtain keys with the same security properties between A–C. A way this can be achieved in practice is as follows: 1. One key is generated on each link (a key $K_{AB}$ between Alice and Bob, and a key $K_{BC}$ between Bob and Charlie); 2. Bob, who holds both keys, encrypts $K_{AB}$ using $K_{BC}$ as a key, with a quantum-safe encryption algorithm which ensures unconditional security, such as One-Time Pad (OTP); 3. Bob then sends this encrypted key to Charlie via Internet, who can decrypt it using $K_{BC}$; 4. Now both Alice and Charlie hold the same key $K_{AB}$, which we will now call $K_{AC}$, and they can use it for further unconditionally secure communication.

This approach achieves the goal of extending the unconditionally secure key exchange between end-users who are not directly connected via a quantum link. However, we can observe two drawbacks: 1. It assumes forwarding nodes (Bob, in this example) are honest (since Bob has access to $K_{AC}$, he can store it and use it to read the encrypted communication between Alice and Charlie); and 2. From the standpoint of network key generation efficiency, two keys are consumed to obtain one. We consider the keys consumed since, with OTP, any re-usage of any of the keys involved would nullify their property of unconditional security and would give valuable information to a potential malicious actor. In practice, key rate efficiency in a QKD network is an important and desired property, since commercially available QKD hardware have limited key generation rates—typically, in the range of 2KB/sec of key bits^2^, due to inherent limitations of quantum channels^4^ (although theoretical improvements have recently been discovered, for example for Continuous Variable QKD^5^). Moreover, the practical implementations of this forwarding mechanism work based on a greedy approach, where the keys are forwarded between any end-users who request it. This means, in our example, Alice may constantly request keys $K_{AC}$, Bob obliges, and as a consequence of key consumption Bob and Charlie are starved of keys $K_{BC}$. This is an impactful problem in practical QKD networks such as EuroQCI, which are federated both at national level (in the case of QKD devices held and maintained by different independent state institutions, non-governmental organizations, even perhaps private entities) and at international level (as is the case of the cross-country links within the European Union, where each country maintains a level of autonomy on its national network). The expected impact of such networks on a global scale is known to be massive, especially in areas of unconditional security, long-distance communication, and reduced computational complexity^6^ . In this work we address the issue of equitable key generation in a complex network, and we provide an algorithm that identifies the optimal key distribution strategy which loses the minimum amount of keys while achieving a distribution goal across one of several scenarios.

Similar approaches using maximal multi-commodity flow linear programming solution have been proposed^7^ for generic QKD networks and in^8^ tailored for space segments trusted repeaters using low-earth orbit and geostationary equatorial orbits. A similar approach^9^ with the same algorithm and maximization metric evaluates the algorithm on two deployed QKD networks (SECOQC and NSFNET). Other QKD routing algorithms have been published in the literature that have other focuses than optimality of key distribution, such as: dynamic routing using a modified OLSR protocol based on link availability^10^, node discovery and iterative routing^11^, hybrid encrypted and unencrypted routing based on path selection and IP address assignment schemes^12^. Other techniques involve multi-party key distribution at the level of the quantum protocol^13,14^ but with less applicability to existing QKD devices. KMS designs for large scale networks^15^ or with focus on secure key forwarding^16^ have been studied before, as well as QKD analysis in light of network topology and performance indicators^17^, but without a practical and scalable approach for key forwarding in the various scenarios we have proposed. Similar linear programming approaches have been used for entanglement distribution^18,19^ and for physical-level wavelength-division multiplexing^20,21^. A thorough description of the architecture, interfacing protocols, and security of a scalable metropolitan network has been presented in the literature^22^. To our knowledge this is the first algorithm providing the optimal solution in the context of the proposed scenarios and the most extensive analysis of the multi-commodity flow-based OTP-secured key forwarding in a QKD network.

## Problem definition and LP algorithm

We model the issue of key rate distribution in a complex QKD network as an optimization problem for which we provide an optimal solution. Our approach involves the following steps: defining the optimization problem and its goal; proposing a list of optimization scenarios with applicability to real-world QKD networks; formalizing the problem and the process of key forwarding; modelling the formalization as an LP problem to ensure optimality; analysing the results and discussing the collected insights by optimization scenario, network topology, and QKD parameters. In the rest of this section we expand on each of the points above.

### Optimization problem and goal

The premise of the problem is a QKD network graph where nodes represent end-users and edges represent QKD links between end-users. Each link has an associated weight (although we will call it weight as is usual in graph terminology, its meaning is that of a profit, not of a cost) which represents the key generation rate available on that link. Key rates may differ across the links of a network due to several factors (to mention a few: different vendors of QKD hardware, different QKD hardware from the same vendor, distance between end-users, environment perturbations).

The QKD infrastructure can be viewed as having several layers: (1) Physical layer: the medium through which quantum information is exchanged (typically fiber optics or free space) together with the physical QKD devices which enable this process. (2) QKD layer: the software running on the QKD devices which continually exchanges and processes quantum information in order to generate secure keys along a link. (3) Vault layer: a database layer which aggregates the generated keys and stores them in long term memory for future use. (4) KMS layer: the key management system layer which handles key usage, including key forwarding across links and interfacing the stored keys with the applications which require them. (5) Application layer: the user-facing applications which make use of keys fetched through the KMS (for example, a secure file transfer application or a quantum-safe VPN solution).

Our protocol is implemented at the KMS level. The KMS will be shipped in practice together with the QKD device by the network administrator at the level of the federated network where key redistribution is desired (hence, the end-user will not have access to manually edit or overwrite the KMS’s key redistribution rules). It is also in the responsibility of the KMS to apply the redistribution according to the scenario selected by the administrators of the network. The reason for this is simple: in a typical QKD network, key generation between non-adjacent nodes is served through a greedy forwarding mechanism as presented in the Introduction section. The forwarding is greedy in the sense that it works on a first-come-first-served approach: if node A sends many requests for keys $K_{AC}$ with node C, and the path from A to C passes through node B, then nodes B and C may inadvertendly be starved of keys $K_{BC}$, whereas perhaps the desire of the network administrators at that time is for a broadcast distribution where the goal is to maximize the number of keys $K_{BX}$ for all *X*. This can be achieved practically by imposing a limit to A on the number of keys she can request with node C so as not to impact the key generation for the target pairs $K_{BX}$ as desired by the network administrators. As such, the network administrators that provide and install the QKD device on the premises of A, will also provide a KMS software that imposes this limit on all nodes according to the optimization goal (which may be one of the scenarios proposed in this paper, but may also be any arbitrary linear constraint on the key rates of some or all of the pairs).

We further extend the network to a complete graph, where the edges that connect end-users who share a QKD link are called “physical links”, and the edges that connect end-users who do not share a QKD link are called “logical links”. A logical link represents the indirect connection between two end-users which passes through at least two physical links. In the rest of this section, we will use the terminology of “link” to refer to a link of any type (physical or logical). We assume the network graph is connected (without loss of generality, in a disconnected graph the protocol may be applied within each connected component independently).

The goal is to maximize the key rate across a given set of target links (as defined in the scenarios section below). The equitability property of redistribution refers to the act of ensuring that key rate is redistributed equitably among the nodes. This is achieved through a careful selection of the metric to maximize: the protocol maximizes the minimum key rate along any of the target links.

The act of key forwarding is modelled as follows. The key forwarding is akin to consuming one key (in the context of balancing key rate, consumption will refer to key rate—e.g. consuming one key per second) from a link A–B and one key from a link B–C in order to produce a key along the link A–C. In terms of KMS rules, in this process the KMS solutions running at A and B need to reserve one key (out of all keys generated between A and B) for usage between A–C; the KMS solutions running at B and C need to reserve one key (out of all keys generated between B and C) for usage between A–C; with this reservation in place, the KMS at B can proceed with the OTP encryption and key forwarding while redistribution balancing is ensured according to the desired scenario. This key reservation mechanism is an essential element of our proposed formalization.

### QKD network balancing scenarios

We define the following main scenarios with practical applicability.

**Figure 1:**
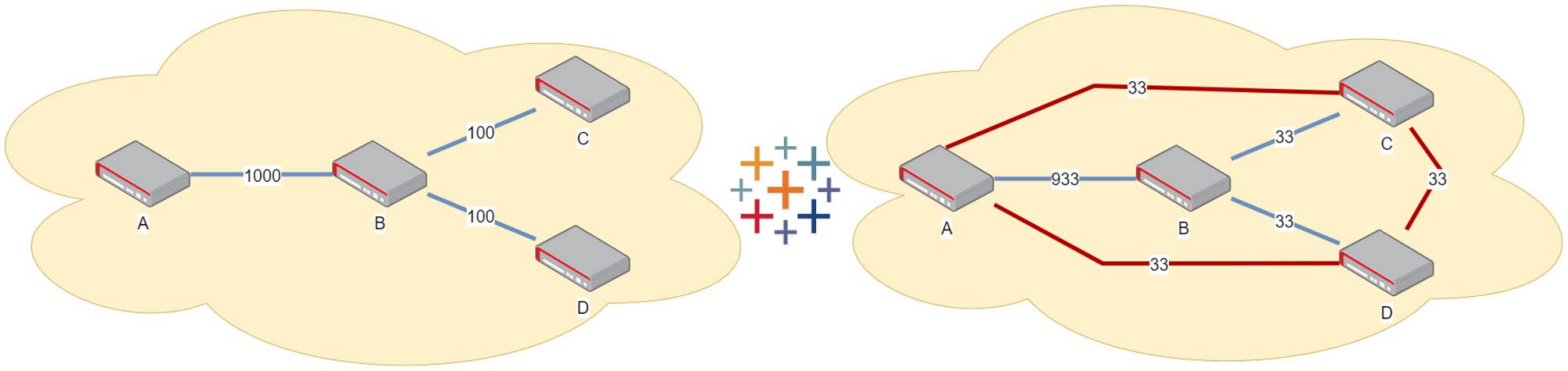
Balanced Scenario: An example of the balanced scenario $S_{A2A}$: a 4-node star network where every pair of nodes is part of the target group. In this graph, links A–B, B–C, and B–D are physical; links A–C, A–D, C–D are logical. The left side of the figure presents the original network configuration; the right side presents the optimal key rate obtained by applying the algorithm configured in the balanced mode. The global minimum key rate has the maximum possible value of 33.

All-to-All (Balanced) Scenario ($S_{A2A}$)—see Fig. 1: this scenario is applicable to a federated QKD network where all end-users are equal and there is no preferential set of nodes. Each end-user would like to have a key rate that is as high as possible with all other nodes, without taking a significant toll on the overall key generation rate of the network. An example of balanced network is provided, where the desired behaviour is to maximize the minimum key rate between any pair of two nodes, either directly connected with a physical link or not (the logical links are displayed in red).

**Figure 2:**
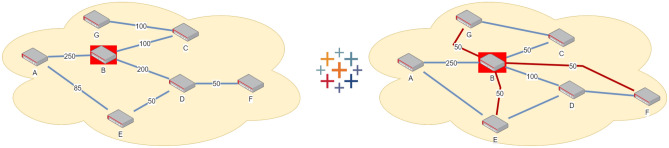
Broadcast Scenario: An example of the broadcast scenario $S_{O2A}$: a 7-node network where the goal is to maximize the key rate exchanged between node B (marked in red) with every other node in the network. Among the target links: B–A, B–C, and B–D are physical links; B–E, B–F, B–G are logical links. The minimum broadcast key rate has the maximum possible value of 50.

One-to-All (Broadcast) Scenario ($S_{O2A}$)—see Fig. 2: this is the scenario where one particular node is preferential and it is desired to maximize the key rate between the preferential node and all other nodes. For example, within a national quantum communication infrastructure, the government may occasionally want to maximize the key rate between its central agency and all other nodes, at the expense of a lower key rate between non-preferential nodes. An example broadcast network is provided, where the desired behaviours is for node B to maximize its minimum key rate with every other node.

**Figure 3:**
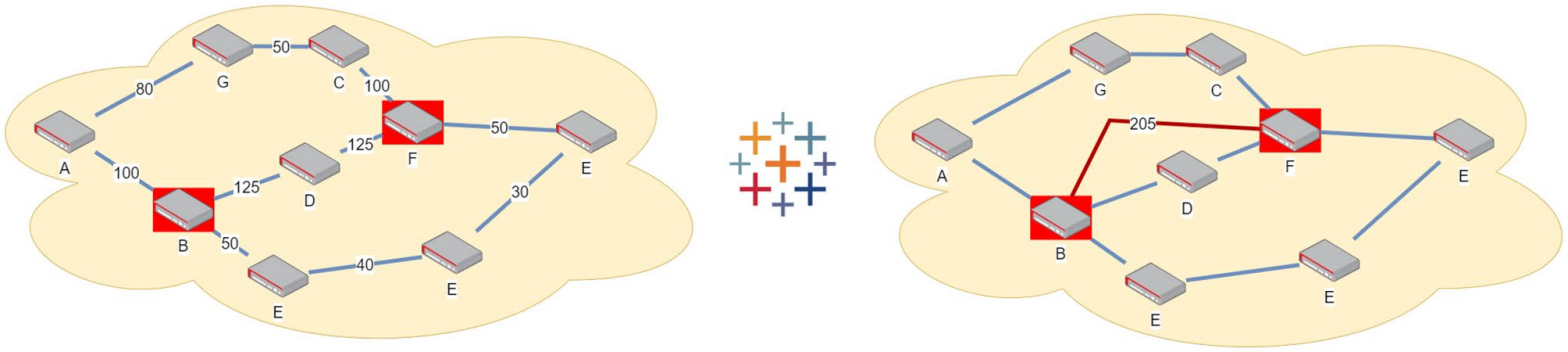
High-throughput Scenario: An example of the high-throughput scenario $S_{O2O}$: a 9-node network where the pair B–F must exchange keys with a rate as high as possible at the expense of the key rate between any other pair of nodes. The obtained key rate between B and F is at the optimal value 205.

One-to-One (High-throughput) Scenario ($S_{O2O}$)—see Fig. 3: a scenario where within a complex network a specific link (either physical or logical) must be prioritized at all costs. For example, in a critical situation (war, natural disaster, etc) real time high-throughput communication is required between first responders and affected areas, at the expense of the communication between any other members of the network. An example of a high-throughput connection is provided, where nodes B and F must achieve the maximum possible key rate at the expense of the communication of any other node pair.

### Problem formalization

The main structure is the network’s complete undirected graph $G = (V, E, w, c)$ where: *V* is the ordered set of vertices, each node corresponding to a QKD end-user; *E* is the set of edges, each edge corresponding to a (physical or logical) link between two end-users; $c: E \rightarrow \{black, red\}$ is the edge color function. The color of the edge represents its type (black edges are physical, red edges are logical); $w: E \rightarrow \mathbb {R}^+ \cup \{0\}$ is the edge weight function. The weight attached to each edge represents the key rate exchanged between the connected nodes. In the input graph, black links have a weight that is equal to the measured continuous key rate between the QKD devices at the ends of the link, while red edges have a weight of 0 (there is no key exchange if nodes are not directly connected physically). After the optimization algorithm is applied, the output consists of a redistribution map for each physical links, as well as the updated weights (i.e. key rate) of both black and red edges as per the scenario and the optimization rules.

According to the desired scenario, a set *T* of target links is selected. For $S_{A2A}$, the target set contains all links in the complete graph: $T = E$. For $S_{O2A}$, the target is the set of all links between the given target node *t* and every other node: $T = \{ (t, u) \,|\, u \in V \text { and } u \ne t \}$. For $S_{O2O}$, the target contains one single link between the two target nodes as defined in the scenario: $T = \{(t_1, t_2) \,|\, t_1 \ne t_2\}$.

We formalize the concept of “balanced” weight redistribution by a careful selection of the metric to maximize: the optimization goal is to maximize the value $n = \min _{\tau \in T} w(\tau )$.

We formalize weight redistribution as follows. For each target $\tau =(t_1, t_2) \in T$, we consider without loss of generality that keys flow (i.e. get forwarded) from $t_1$ to $t_2$ if $I_{t_1} < I_{t_2}$ where $I_v$ is the index of node *v* in *V*. Keys may flow from $t_1$ to $t_2$ along any path in $P = \{ \text {path from } t_1 \text { to } t_2 \}$ that passes only through *black* edges. Assume that in (one of) the optimal distribution(s), *k* bits of key rate flow from $t_1$ to $t_2$ along a path of length *L* $p = \{(t_1, v_1), (v_1, v_2),..., (v_{L-1}, t_2)\}$. Since key forwarding implies the key bits cannot be re-used by any of these links, this is equivalent to reserving *k* key rate bits in each of the links that are part of path *p* specifically for the target $\tau = (t_1, t_2)$. As such, we define the redistribution function $r: T \times E_{black} \rightarrow \mathbb {R}^+ \cup \{0\}$ which maps each combination of target $\tau \in T$ and physical edge $e \in E \,\text {such that}\, c(e)=black$ to the number of key bits per second generated along *e* that are reserved for target $\tau$.

To align the output of the algorithm with the practical requirements of a KMS, we further define weight redistribution based on nodes rather than edges. Consequently, for each node *v* we define two distribution functions $r^v_{in}: T \times \{(v, u) \in E_{black}\} \rightarrow \mathbb {R}^+ \cup \{0\}$ and $r^v_{out}: T \times \{(v, u) \in E_{black}\} \rightarrow \mathbb {R}^+ \cup \{0\}$ for the forwarding of keys through *v* from and to neighboring nodes, with the important property of key conservation per node: $\forall \tau \in T \sum _{(v,u) \in E_{black}} r^v_{in}(\tau , (v,u)) = \sum _{(v,u) \in E_{black}} r^v_{out}(\tau , (v,u))$. Obviously, for any target $\tau = (t_1, t_2)$, $t_1$ is a key source ($r^{t_1}_{in}(\tau , (t_1, u)) = 0 \; \forall u$), while $t_2$ is a key drain ($r^{t_2}_{out}(\tau , (t_2, u)) = 0 \; \forall u$). Additionally, for key conservation per edge we need to impose the rule that $r^v_{in}(\tau , (v,u)) = r^u_{out}(\tau , (v,u)) \; \forall v, u \in V, v \ne u$.

In short, the goal of the present research is to provide an optimal set of functions $r^v_{in}$ and $r^v_{out}$ for every node *v* which maximize the metric *n* along the set of targets as selected in *T*. We stress the fact that *T* may contain any combinations of links in *E*, and the analysed scenarios $S_{A2A}$, $S_{O2A}$, and $S_{O2O}$ are only defined for more accurate comparison with practical situations in QKD networks, not as limitations of the algorithm.

### LP problem statement

Following the formalization presented above, the KMS-level key distribution is equivalent to the fractional multi-commodity flow problem, where multiple commodities (keys between any of the target pairs $\tau =(t_1,t_2)$) need to flow in a graph (more specifically, the network subgraph composed only of *black* physical links) between a source (the node $t_1$) and a drain (the node $t_2$) where each link has a maximum flow capacity (the key rate *w*(*e*)). The multi-commodity flow problem is known to be NP-complete for the discrete case (i.e. where commodity flows in any given link are integers), but with fractional flows the problem can be solved optimally in polynomial time with linear programming^23^. Even faster approximation schemes may be employed^24,25^. The fractional approach can be used in this case because we consider the key rate is measured in key bits per second; the meaning of fractional redistribution is that a number of key bits must be reserved along a time window longer that one second.

Linear programming is a modelling technique for maximizing (or minimizing) the value of an objective function linear in any number of lower bounded parameters based on a list of linear constraints (equations and inequalities) on said parameters. The list of linear constraints define a multidimensional convex polytype over which the function is optimized, which is proved to guarantee optimality.

To state the problem as an LP problem, we consider the universe set $U = \{(e_t, (u, v), d) | e_t \in E, (u, v) \in E_{black}, u \ne v, d \in \{in, out\}\}$ with $e_t = (t_1, t_2) \in N$ representing any potential target (to account for commutativity of target ends, we consider the target $(t_1, t_2)$ as an ordered pair of nodes such that $t_1 < t_2$), (*u*, *v*) representing an ordered pair of nodes connected via a physical link that are used as part of the key forwarding for the target $e_t$, and *d* representing the direction of the forwarding (*in* meaning from *v* to *u*, *out* meaning from *u* to *v*). We associate to every element $((t_1, t_2), (u, v), d)$ of universe *U* the LP variable named t1_t2_u_v_d. Additionally, we define the variable n as the goal to maximize.

Converting the rules as defined in the formalization section into LP formalism following the multi-commodity flow problem’s solution, we obtain the constraints in Algorithm 1 (marked C in the pseudocode). The meaning behind each constraint type is explained below:

**Algorithm 1:**
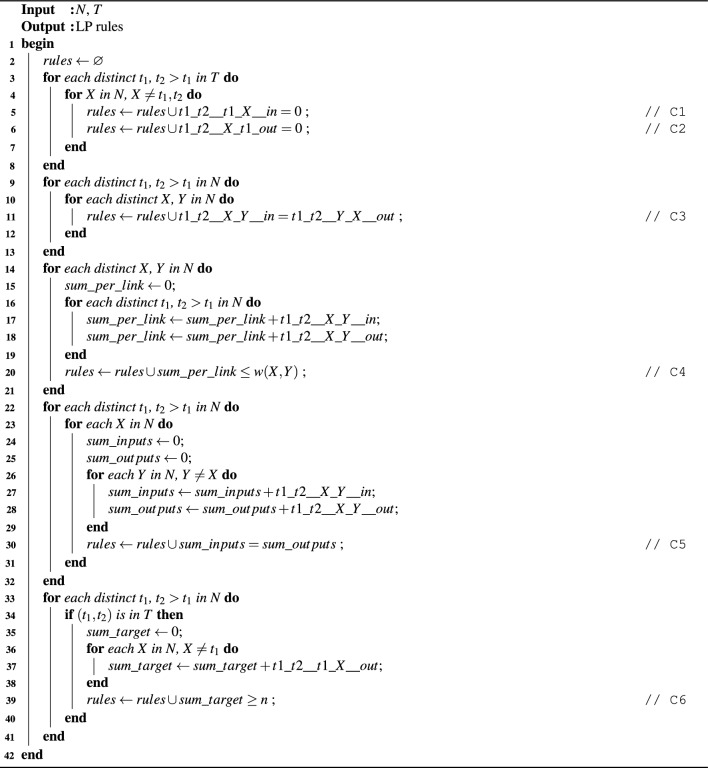
Generating the LP rule set

- C1: if $(t_1, t_2), t_2>t_1$ is a potential target, then $t_1$ is a key source (it does not have any redistribution inputs)
- C2: if $(t_1, t_2), t_2>t_1$ is a potential target, then $t_2$ is a key drain (it does not have any redistribution outputs)
- C3: if $\tau = (t_1, t_2)$ is a potential target and (*X*, *Y*) is a link through which redistribution for $\tau$ may potentially be done, then the input of node *X* from node *Y* for target $\tau$ is equal to the output of node *Y* to node *X* for target $\tau$ (flow reversibility)
- C4: for every link (*X*, *Y*), the sum of all key forwarding rates along this link regardless of target must not exceed the physical key rate capability of the link
- C5: for every potential target $\tau = (t_1, t_2)$ and every node *X*, the sum of all inputs of *X* for target $\tau$ must be equal to the sum of all outputs of *X* for target $\tau$ (key conservation along flows from $t_1$ to $t_2$)
- C6: for every potential target $\tau = (t_1, t_2)$, if $\tau \in T$ according to desired scenario then we impose the goal that the resulting key rate between nodes $t_1$ and $t_2$ must equal or exceed the metric n to be maximized (as this is the optimization goal: maximizing the minimum key rate between the nodes of any target pair).

### Simulation

In order to measure the impact of network topology and size, we have generated two sets of random networks: set A (to assess impact of network size): 15,000 random networks (5000 per scenario), each with a number of nodes uniformly sampled from [3,40] and a number of redundant edges uniformly sampled from [0, $max_E = min(15, \frac{\frac{|V|*(|V|-1)}{2}-(|V|-1))}{2}))$] (the limit at 15 is for limiting the computation time for larger graphs); and set B (to assess impact of network topology): 1250 random networks, each with exactly 15 nodes and 7 redundant edges. Here, |*V*| is the number of nodes in the network and $max_E$ is the number of redundant edges that would need to be added to make the graph complete. Since each redundant edge added creates an independent cycle, the number of redundant edges is also equal to the cyclomatic number of the generated network. The generation method for a random graph with *N* nodes and *e* redundant edges is as follows: (1) Create initial set of vertices *V* which contains 1 vertex, and the initial (empty) set of edges *E*; (2) For *i* from 2 to *N*: select a random vertex *w* from *V*, add an additional vertex *v* to *V*, and append a new edge (*v*, *w*) to *E* to ensure the graph is connected; (3) Create a list *L* with all pairs $(v_1,v_2)$ from *V* that do not share an edge; (4) Uniformly sample *e* elements from *L* and append them to *E*. To ensure an accurate analysis of network size and topology which is not caused by differences in key rates across networks, all simulations have been performed with all links of equal key rate (100 keys/second).

## Performance analysis

**Figure 4:**
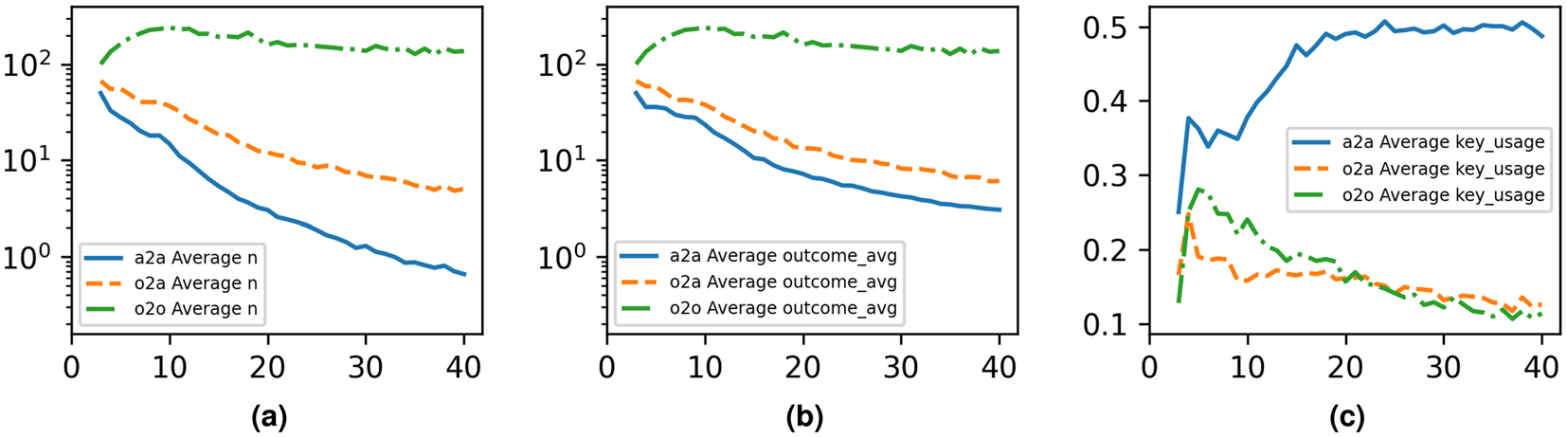
The evolution of performance parameters as the network size increases along 15,000 runs on randomly generated graphs of 3–40 nodes with 0–15 redundant links. (**a**): Optimal *n* maximized in a random graph network by number of nodes. X Axis: Number of nodes; Y Axis: Average optimal *n*. (**b**): Evolution of average outcome key rate exchanged between all node pairs $\tau$ in *T*. X Axis: Number of nodes; Y Axis: Average key rate. (**c**): Evolution of key consumption for target optimization as a percentage of initial total network-generated key rate by number of nodes. X Axis: Number of nodes; Y Axis: Key consumption as a percentage of total.

We analyze the resulting optimal key rate redistribution which achieves the highest minimum *n* across every target node pair. The analysis is performed in three different scenarios with practical applicability: all-to-all (every pair of nodes in the network is a target), one-to-all (all pairs made out of a specific fixed node and every other node), and one-to-one (one specific pair is the only target).

We plot in Fig. 4a the evolution of the optimal *n* as the network size increases. We observe a significant drop in the optimal *n* as network size increases, which is expected since the number of target pairs increases with network size linearly in the one-to-all scenario and quadratically in the all-to-all scenario. For the one-to-one scenario, we observe a significant increase above the initial physical link key rate (100), and then a slight decrease as the network size increases which is due to the decreasing ratio of cycles to nodes (because cyclomatic number is limited at 15). In Fig. 4b we plot the evolution of the average outcome key rate between all node pairs, defined as the mean across the 15,000 experiments of the average key rate exchanged between all pairs within one experiment. In Fig. 4c we plot the evolution of key usage for the all-to-all scenario. In one-to-all and one-to-one scenarios, the algorithm is designed to optimize the targets at the expense of key rates within the rest of the network, while in the all-to-all scenario the key usage is (not explicitly) part of the optimization goal. We define key usage as $1-K_g$ where key gain, $K_g$, is the ratio between the resulting total usable key rate generated between any pair of nodes in the network and the initial total key rate of all physical links. We expect the key consumption to go up as the number of nodes in the graph increase since larger network size implies more redistribution and along longer routes; however, the key consumption appears to converge to 50% on average.

**Figure 5:**
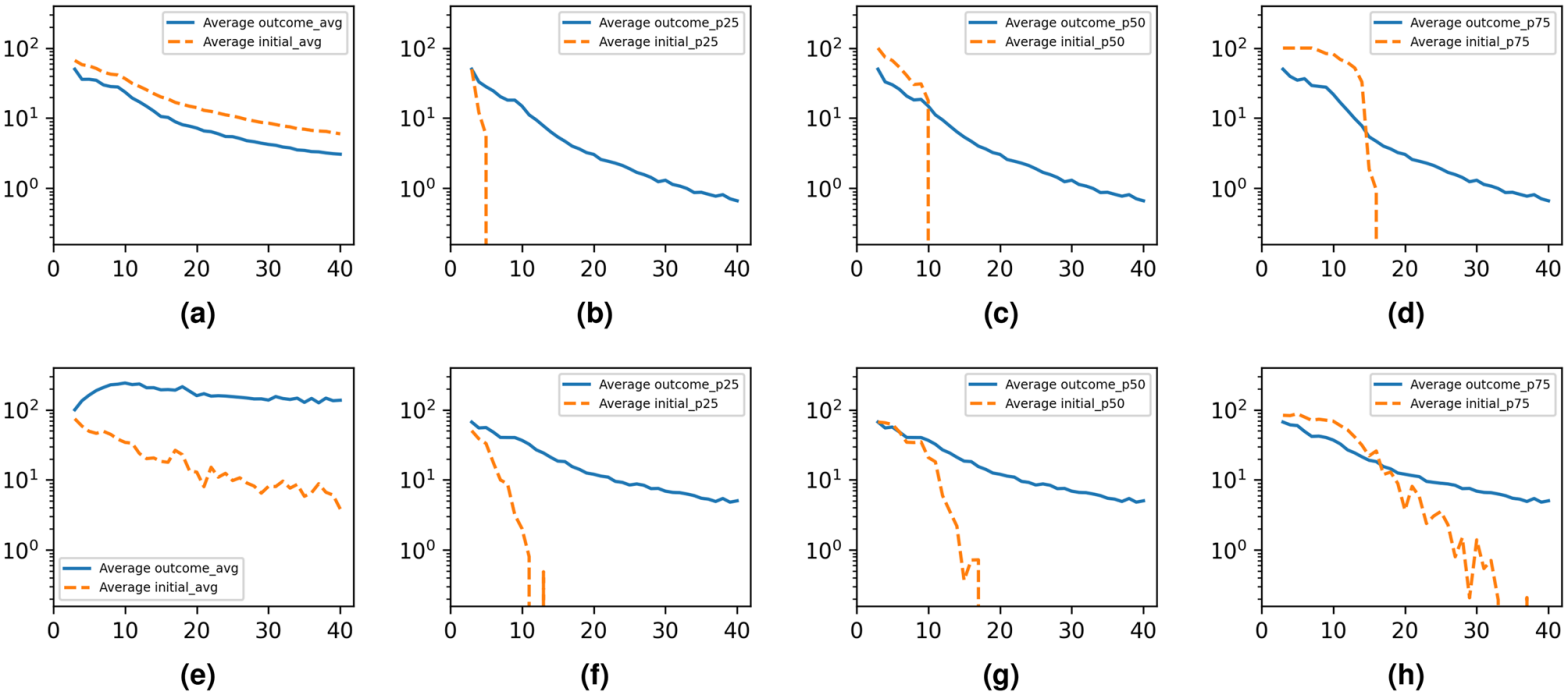
The evolution of key rates between target nodes by number of nodes along 5000 runs per scenario on randomly generated graphs of 3–40 nodes with 0–15 redundant links, plotted with the initial key rates for comparison. For the $S_{A2A}$ scenario, average outcome key rate between targets and average intial key rate between targets for (**a**): all $S_{A2A}$ runs, (**b**): top 25% percentile of key rates in $S_{A2A}$ runs. (**c**): top 50% percentile of key rates in $S_{A2A}$ runs (**d**): top 75% percentile of key rates in $S_{A2A}$ runs. (**e**): For the $S_{O2O}$ scenario, the average key rate between the two target nodes. For the $S_{O2A}$ scenario: (**f**): top 25% percentile of key rates; (**g**): top 50% percentile of key rates; (**h**): top 75% percentile of key rates.

In Fig. 5 we analyze the distribution of key rates between all pairs of nodes that are part of the target set, both in the initial graph and in the outcome resulting from the application of the algorithm. We plot the average key rate (Fig. 5a), and the 25%, 50%, and 75% percentiles (Fig. 5b–d) for the all-to-all scenario. For the one-to-one scenario we plot the key rate (within each experiment the target is a single link so there is no reason to define percentiles) in Fig. 5e. For the one-to-all scenario we plot the 25%, 50%, 75% percentiles (Fig. 5f–h). We observe a significant drop in the average key rate exchanged for the all-to-all scenario (which is expected since key rate flows from the initial physical links to the desired target pairs while consuming keys), but an eventual significant increase in the percentile distribution of key rates (which rapidly hits 0 with the network size in an unprocessed graph). The average key rate obtained in the one-to-one scenario exceeds one order of magnitude improvement over the average of the initial graph, which is explainable due to all nodes providing their entire key rate bandwidth for the single target. In this case, the obtained key rate between the two target nodes will always be at least equal to one physical link’s key rate (since the nodes are connected, there is at least one path between them and all nodes along the path will provide the entire bandwidth); higher key rates are obtained whenever there are multiple paths between the target nodes that are not bottlenecked by the same edges. To further understand the roll-off to 0 of the 25, 50, and 75 percentiles averages, consider the methodology for computing these values as follows: (1) For a given graph, we list all the target pairs that the algorithm aims to optimize (for example, in the Balanced scenario $S_{A2A}$, the list would contain all possible pairs of non-identical nodes); (2) We sort the list ascending by key rate (initially, non-adjacent nodes would have a key rate of 0, since no key forwarding has been performed); (3) We compute the 25, 50, and 75 percentile values for this list; (4) We perform steps 2 and 3 twice: for the initial graph and key rates, and for the outcome graph and key rates after applying the balanced key reservation and forwarding; (5) We repeat steps 1–4 for a large number of randomly generated graphs, and we average the values. Thus, the roll-off to 0 of the 25/50/75 percentiles of key rates for some given scenario starting at some number of nodes N means that the bottom 25%/50%/75% of all target pairs within that scenario have a key rate of exactly 0. This is expected, since with larger number of nodes the percentage of target pairs which are not adjacent grows (unless assuming fully connected graphs only, which do not have significant practical utility due to prohibitive costs of QKD links).

**Figure 6:**
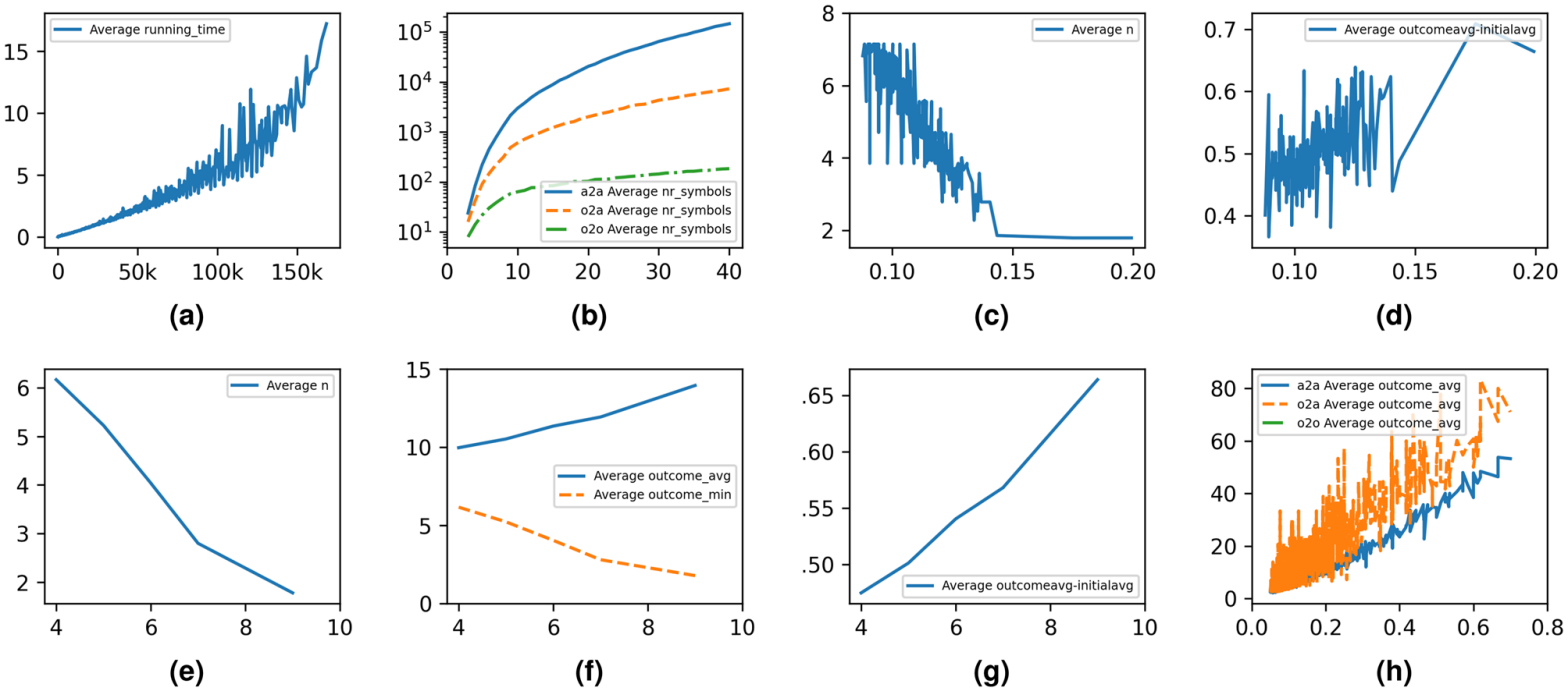
Analysis of algorithm complexity and discussion of performance metrics by network topological factors. (**a**): Algorithm running time by number of symbols in the LP problem statement. (**b**): Evolution of number of LP symbols by network size per scenario. (**c**): Optimal *n* by graph betweenness centrality in $S_{A2A}$ scenario. (**d**): Ratio of average outcome target key rate and average initial key rate by graph betweenness centrality in $S_{A2A}$ scenario. (**e**): Optimal *n* by graph diameter. (**f**): Average outcome target key rate and optimal *n* by graph diameter. (**g**): Ratio of outcome average key rate to initial average key rate by graph diameter. (**h**): Average outcome key rate by graph degree centrality per scenario.

### Complexity analysis

In Fig. 6a we plot the algorithm running time in seconds by the number of LP variables. While a polynomial or exponential relationship is to be expected, the plotted results appear close to linear which can be explained by the numerical stability and sparsity of the problem: most variables are 0, because the paths between any two nodes are likely not to pass through most of the rest of the nodes. In Fig. 6b we plot the number of LP variables by the network size per scenario, and we observe it is proportional to $|V|^2$ for the one-to-one scenario (two symbols per node pair), to $|V|^3$ for the one-to-all scenario (two symbols per node pair per target, where the number of targets is proportional to number of nodes), and to $|V|^4$ for the all-to-all scenario (two symbols per node pair per target, where the number of targets is proportional to number of nodes squared).

### Impact of network topology

For accurate analysis by network topology, we generated 1250 graphs with 15 nodes and 7 redundant links each, within the all-to-all scenario. We analysed several graph topology metrics: diameter (longest shortest path), radius (minimum eccentricity), average path length, density (ratio of number of edges to possible edges, which is constant in this experiment), degree centrality (indicates overall inter-connectivity of the network), closeness centrality (indicates overall node proximity of the network), and betweenness centrality (indicates overall level of bottlenecks of the network), the most remarkable of which are plotted in Fig. 6c–h. The results indicate that the more close together a graph is (smaller diameter, fewer bottlenecks; in the extreme case, a fully-connected graph), the higher the optimal *n*. On the other hand, the more spread apart a graph is (larger diameter, more bottlenecks; in the extreme case, a line graph where the path between the most extreme nodes passes through all other nodes), the lower the optimal *n*. This observation is easily explained by the fact that larger diameter implies more pairs of nodes for which the redistribution flows through the same bottleneck paths. Considering networks with no redundant links (i.e. trees), a graph with smaller diameter (for example, a star topology with a single central node connected to all others) may produce a higher key throughput in the balanced and broadcast scenarios, whereas line-like topologies may cover a larger terrestrial area, at the expense of lower optimal *n* in these scenarios. Another observation is that the outcome average key rate between the targets increases when the optimal *n* decreases, since lower redistribution also means lower key consumption rate, increasing the average key rate exchanged.

## Conclusion

In this work we introduce the relevant concepts in QKD-generated keys secure forwarding using OTP and motivate the need for this given the security requirements and the low key rate of commercially available QKD devices. We introduce the graph mathematical formalism that we use to model QKD networks and to extend the network graph to the complete graph using logical links between all nodes that are not physically connected with QKD infrastructure. We provide a multi-commodity flow statement of the problem, and three scenarios with practical applicability in typical QKD use-cases. We give a description in LP syntax which we run and analyze on 16,250 total simulated networks of up to 40 nodes and 15 redundant links, providing a thorough investigation on the algorithm results and performance as well as the impact of graph size and topology.

As future work, we note that with this approach we can tackle any kind of QKD network key forwarding problem in the same formalism, including optimal addition of QKD physical links and the generation of goal-oriented time-based forwarding schedule.

## Data Availability

The simulation code and the datasets generated during the current study will be made available from the corresponding author upon reasonable request.
